# Occurrence of *Escherichia coli* and faecal coliforms in drinking water at source and household point-of-use in Rohingya camps, Bangladesh

**DOI:** 10.1186/s13099-019-0333-6

**Published:** 2019-11-01

**Authors:** Zahid Hayat Mahmud, Md Shafiqul Islam, Khan Mohammad Imran, Syed Adnan Ibna Hakim, Martin Worth, Alvee Ahmed, Shanewaz Hossan, Maliha Haider, Mohammad Rafiqul Islam, Ferdous Hossain, Dara Johnston, Niyaz Ahmed

**Affiliations:** 1 International Centre for Diarrhoeal Disease Research, Bangladesh, Dhaka, 1212 Bangladesh; 2 WASH Division, UNICEF Bangladesh, Dhaka, Bangladesh; 3 Laboratory of Environmental Health, Laboratory Sciences and Services Division, icddr,b, 68, Shaheed Tajuddin Ahmed Sarani, Mohakhali, Dhaka, 1212 Bangladesh

**Keywords:** Rohingya camps, Drinking water contamination, Point of use water, Faecal coliforms contamination, *E. coli* risk categories

## Abstract

**Background:**

Safe water is essential for life but unsafe for human consumption if it is contaminated with pathogenic microorganisms. An acceptable quality of water supply (adequate, safe and accessible) must be ensured to all human beings for a healthy life.

**Methods:**

We collected and analyzed a total of 12,650 drinking water samples, for the presence of *Escherichia coli* and faecal coliforms, from a large habitation of the displaced Rohingya population comprising of about 1.16 million people living within 4 km^2^.

**Results:**

We found that 28% (n = 893) water samples derived from tubewells were contaminated with faecal coliforms and 10.5% (n = 333) were contaminated with *E. coli*; also, 73.96% (n = 4644) samples from stored household sources (at point of use—POU) were found contaminated with faecal coliforms while 34.7% (n = 2179) were contaminated with *E. coli.* It was observed that a higher percentage of POU samples fall in the highest risk category than that of their corresponding sources.

**Conclusions:**

From our findings, it appears that secondary contamination could be a function of very high population density and could possibly occur during collection, transportation, and storage of water due to lack of knowledge of personal and domestic hygiene. Hence, awareness campaign is necessary, and the contaminated sources should be replaced. Further, the POU water should be treated by a suitable method.

## Introduction

An estimated 1.16 million Rohingya people originally displaced from Myanmar have been living in 32 camps in Cox’s Bazar district of Bangladesh. Newly arrived Rohingyas are living in spontaneous settlements, and there is an increased demand for humanitarian assistance, including shelter, clean water, and sanitation. Collectively, a total of 6057 water points and 50,087 emergency latrines have been built to support the needs of inhabitants of the camps. Out of a possible danger of cholera outbreaks, the Government of Bangladesh and other humanitarian and aid organizations and their partners have immunized 900,000 adults and children against cholera and screened approximately 263,000 children for malnutrition (https://www.unicef.org/emergencies/bangladesh_100945.html). In a challenging hilly terrain of Cox’s Bazar, the unplanned and unprompted way of the settlement of Rohingyas at a very high density has created an unprecedented challenge for water, sanitation, and hygiene (WASH) needs [1]. Under-nourished and stressed populations such as the displaced Rohingyas could be highly predisposed to the possibility of acute watery diarrhea and other water-borne diseases [2].

Water is unsafe for human consumption when it is contaminated with pathogenic microorganisms, and an acceptable quality of water supply must be ensured for all. The prevalence of water-borne diseases including diarrhea, cholera, typhoid fever, and dysentery, has been mainly attributed to unsafe water and unhygienic practices [3–5]. Faecal contaminants going into the water supply could lead to a serious form of water contamination leading to the transmission of enteric pathogens such as *Salmonella* spp., *Shigella* spp., *Vibrio cholerae*, and *E. coli*. These pathogens are usually found in human and animal feces and could possibly reach the sources of community water supply through leaching or other means such as improperly treated sewage [6]. WHO [7] has developed a classification and color-code scheme for *E. coli* colonies per 100 mL water sample. Any potable water may be contaminated microbiologically due to insufficient sanitation and unhygienic practices [8]. In order to estimate the number of microbes present and to find out microbial types, different microbiological water analysis methods are used in different labs. It is a very expensive and strenuous procedure to examine all the possible microbial pathogens in water, and therefore, a specific group of microorganisms that come from the same source as human pathogens is used to indicate the presence of pathogens. In order to indicate the presence of faecal contamination in water, indicator microorganisms were approved for the studies of coliform bacteria in the U.S. Public Health Service in 1914 [9]. If indicator microorganisms are observed in a substance, it designates the presence of faecal contamination and therefore, pathogenic microorganisms might be present in that water.

Samples collected and tested before and after decontaminating the mouth of the tubewells should provide a real scenario to infer if the source of contamination is from water aquifer or the mouth of the tubewell. Usually, the deep underground aquifer is a good source of drinking water which does not require treatment [10], but during water collection, carriage, storage, and use, secondary contamination may occur from the user due to lack of proper knowledge and awareness of hygienic practices [11]. The quality of water stored in households might provide the indicators of the level of secondary contamination by comparing the levels with that of the source water. Such a comparison would also be very important to model household transmission dynamics to understand pathogen flow pathways and to provide proper interventions [12–14]. In Cox’s Bazar, since the Rohingya population is living in a small and highly crowded area and since a huge number of people are using a single water point (on an average, 192 people are getting water from a single tubewell), it is difficult to testify water quality from every household. Therefore, one of the aims of this study was also to analyze the microbiological quality of water source before and after decontaminating the mouth of the tubewell in order to determine the contamination scenario of the aquifer. Further, it was also important to analyze the quality of household water samples relevant to the corresponding source (tubewells) to assess the overall situation of secondary contamination and to get the passive idea about awareness and knowledge gap entailing proper hygienic practices of the Rohingya camp inhabitants.

## Results and discussion

### Point-of-use water is far more contaminated than that of its source

**Fig. 1:**
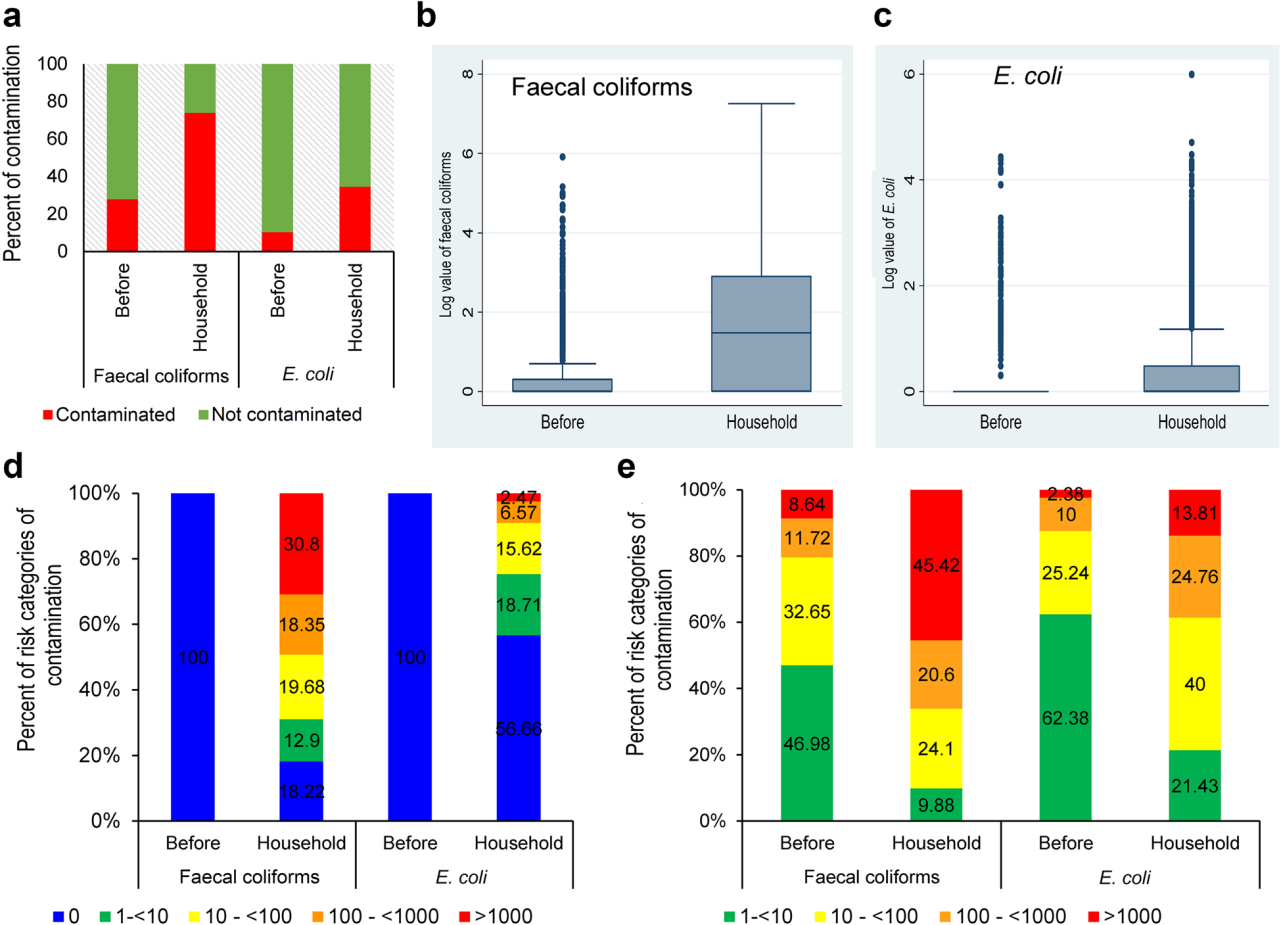
Contamination scenario of faecal coliforms and *E. coli* in source and point of use water samples. **a** Bar diagram showing percent contamination of faecal coliforms and *E. coli*, before decontaminating the mouth of the tubewell and household (POU) water. **b** Box and whisker plot of log value of faecal contamination before decontaminating the mouth of the tubewell and POU water. **c** Box and whisker plot of log value of *E. coli* before decontaminating the mouth of the tubewell and POU water. **d**, **e** Bar diagram showing percent of risk categories of contamination of faecal coliforms and *E. coli*, before and after decontaminating the mouth of the tubewell and POU water

A total of 3186 tubewells were tested for faecal coliforms and *E. coli,* and we found that 28% (n = 893) were contaminated with faecal coliforms and 10.5% (n = 333) with *E. coli* (Fig. 1a). The contamination levels of household point-of-use (POU) water samples were far worse: a total of 6278 samples were tested, and 73.96% (n = 4644) were found contaminated with faecal coliforms and 34.7% (n = 2179) with *E. coli* (Table 1 and Fig. 1a). Other studies also showed that POU water(s) are highly contaminated than those of their sources [8, 15]. It is well established that faecal coliforms can survive in water for longer periods than *E. coli*, so this leads to the thinking that the sources that are contaminated with faecal coliforms and not with *E. coli* might not be contaminated recently. *E. coli* is considered as the indicator of recent faecal contamination, and selection of *E. coli* is common because it is economical to detect and often present where faecal contamination is a problem [7]. *Bacteroides* spp. which is now being used as an experimental indicator of human faecal contamination and considered more reliable than *E. coli* as well as being species specific, is considerably more expensive [16, 17].

**Table 1:** Frequency distribution of faecal coliforms and *E. coli* in water samples from all the tube wells (Source) and households (POU)

	Faecal coliforms			*E. coli*		
	Before	After	Household	Before	After	Household
No. of obs.	3186	3186	6278	3186	3186	6278
Mean (SD)	552.52 (14,773.02)	343.17 (12,027.65)	16,397.2 (253,386.2)	46.73 (877.78)	23.21 (584.14)	253.53 (12,413.4)
Range (min, max)	0, 800,000	0, 700,000	0, 18,000,000	0, 27,000	0, 24,000	0, 980,000
Geometric mean (SD of log variables)	19.88 (1.02)	22.23 (0.98)	197.51 (1.56)	9.58 (0.432)	14.29 (0.317)	16.33 (0.795)

As the range of data is very high, to understand contamination scenario of source and POU water samples, we carried out the box and whisker plot analysis. As shown in Fig. 1b, c, the interquartile ranges in the POU water samples are high as compared to the source water. It appears that the data from source water samples before contamination contains heavy tails and a large number of outliers for both faecal coliforms and *E. coli*. Because of high spread and zero inflation of data, in descriptive statistics, geometric mean is calculated instead of the arithmetic mean (Table 1). These data, therefore, indicate that the household water samples are more frequently found to be contaminated.

The odds of being contaminated by faecal coliforms in POU water are 7.42 times relative to source water (Table 2). On the other hand, the odds of POU water being contaminated by *E. coli* are 4.68 times than that of its source water (Table 2). We have determined and compared the risk-of-drinking of water from a contaminated source to non-contaminated source, and it was observed that the contamination rate increased in the highest risk category wherein the source was contaminated (Fig. 1d, e). It can be assumed that if the source water is not contaminated, then household water becomes contaminated due to unhygienic practices. Of note, our data suggest that when the source water is contaminated, then higher numbers of POU water samples could be graded in the highest risk category, which eventually increases the risk of infection many folds. In this study, only the source and POU water quality were monitored but how the POU water was contaminated was not determined. The contamination of POU could have multiple origins; from the vessels, or from the source water or could occur due to the unhygienic practices of the user. Nevertheless, the contamination of POU water is manifest, and the people are drinking it. Therefore, the determination of the source of contamination is highly important to target intervention, such as cleaning the vessels or treat the water with chlorination. In a challenging environment like Rohingya camps with limited resources, it is recommended to build awareness of hygienic practices as well as provide interventions such as chlorination of POU water to ensure safe drinking water. Although illiteracy or lack of formal education are barriers to understanding, awareness can be enhanced following the interventions, but an actual change of behavior could often be low. Such awareness build up requires a long term effort, but short term interventions including on-site household decontamination are urgently required (chlorination, local low-cost UV purification units, etc.).

**Table 2:** Odds ratio of point of use water comparing contamination with source

Predictors	Category	Odds ratio	Sig.	P-value	95% CI for odds ratio	
					Lower	Upper
Faecal coliforms	Source (ref)		0.01	< 0.01		
	Point of use	7.42	0.3		6.857	8.02
*E. coli*	Source (ref)		0.006	< 0.01		
	Point of use	4.68	0.29		4.14	5.30

### Camp-wise contamination scenario of faecal coliforms and *E. coli* in the Rohingya camps

**Fig. 2:**
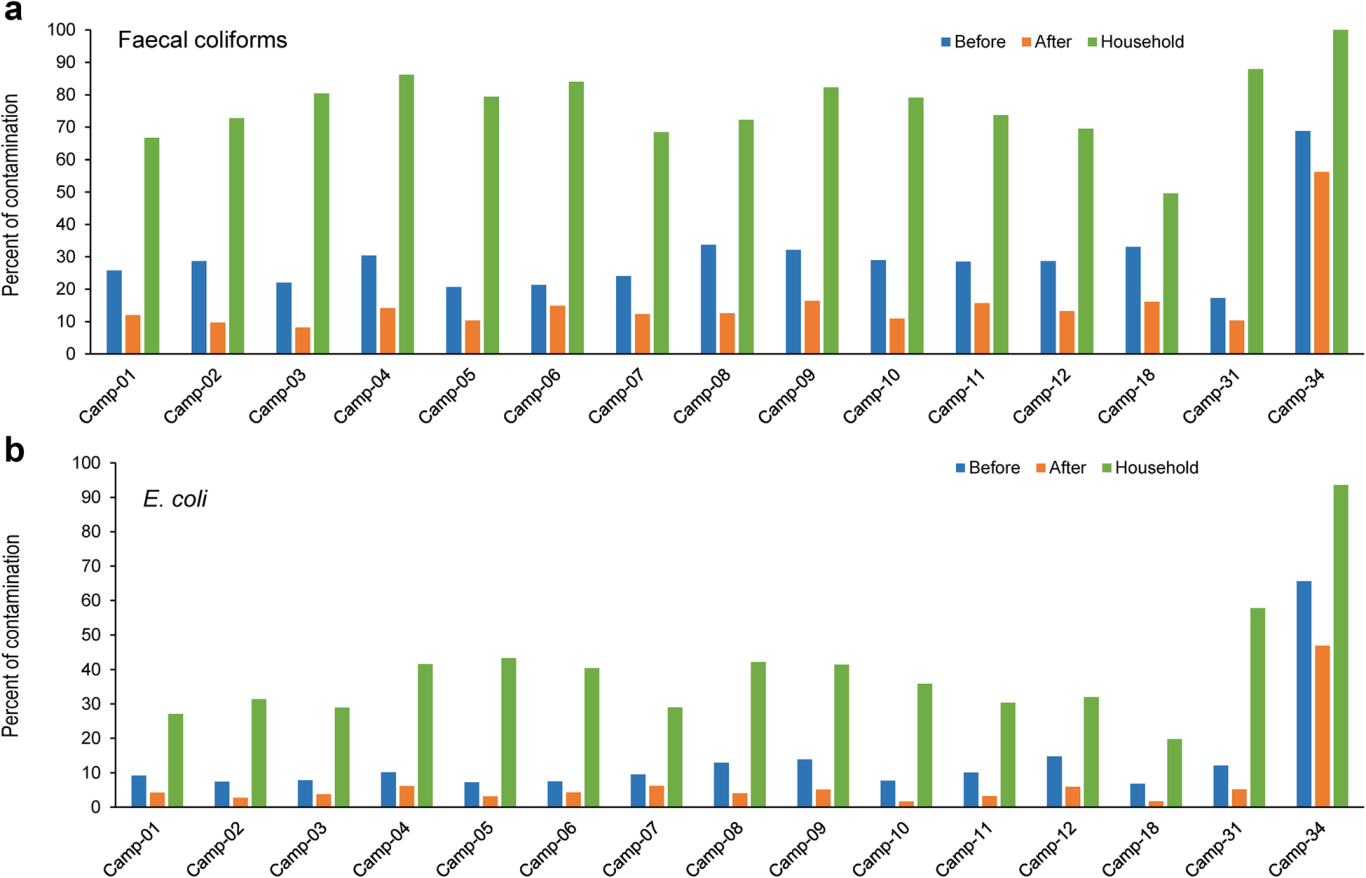
Camp-wise contamination scenario of faecal coliforms and *E. coli* in source and household (POU) water samples. **a** Bar diagram showing percent contamination of faecal coliforms before and after decontaminating the mouth of the tubewell and POU water. **b** Contamination scenario of *E. coli* before and after decontaminating the mouth of the tubewell and POU water. 15 out of 21 camps’ data are shown in the bar diagrams

Camp-wise analysis of contamination data (Additional file 1: Table S1) revealed that greater than 80% of POU water samples from camps 3, 4, 6, 9, 31, and 34 was contaminated with faecal coliforms whereas, 93.5% of POU water of only camp 34 was contaminated with *E. coli* (Fig. 2a, b). Contamination distribution in the camps was similar for both faecal coliforms and *E. coli* irrespective of sample types. There was no significant difference among the camps in case of both faecal coliforms and *E. coli* contamination for all sample types (Fig. 2a, b).

### Most of the aquifers are safe: decontaminating the mouth of the tubewell reduced the source water contamination significantly

**Fig. 3:**
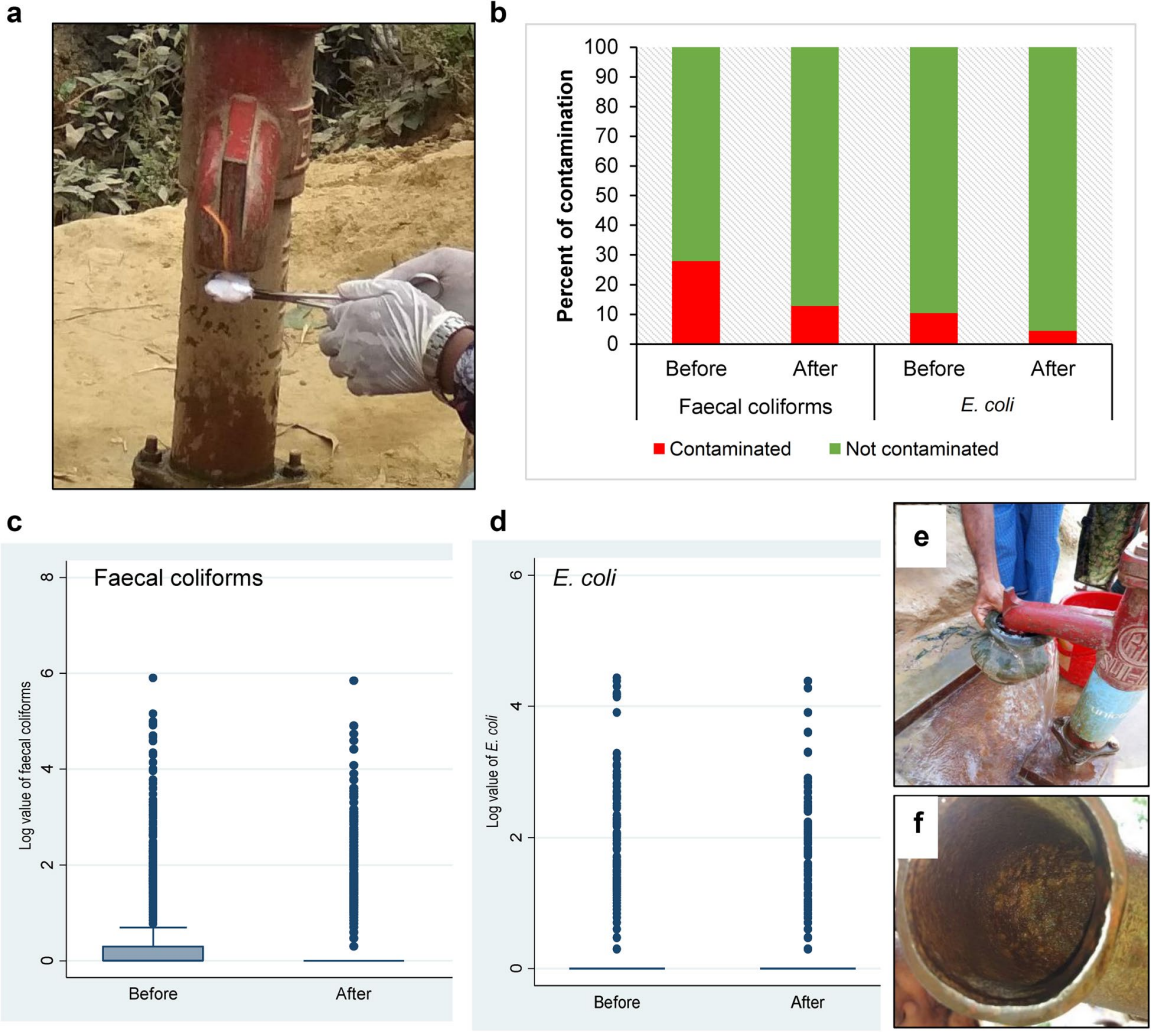
Contamination scenario of faecal coliforms and *E. coli* in source before and after decontamination. **a** Image showing method of decontamination of the mouth of the tubewell by burning with alcohol. **b** Bar diagram showing percent of contamination of faecal coliforms and *E. coli*, before and after decontaminating the mouth of the tubewell. **c** Box and whisker plot of log value of faecal coliforms before and after decontaminating the mouth of the tubewell. **d** Box and whisker plot of log value of *E. coli* before and after decontaminating the mouth of the tubewell. **e** Image showing way of contaminating the mouth of the tubewell by unhygienic practices. **f** Image showing the rough surface of the tubewell mouth and inside of the tubewell mouth

To find out the contamination status of the aquifer, we performed a specific method of collecting water samples after burning the mouth of the tubewells (Fig. 3a) which helped determine if the contamination is coming from the mouth of the tubewell or the aquifer. In Fig. 3b, a multiple bar diagram presenting the percentage of faecal coliforms and *E. coli* contamination of water samples from source (before and after decontamination), clearly states that after applying the decontamination process, the bacterial count decreased in tubewell water.

Box and whisker plot analysis revealed huge numbers of outliers for both faecal coliforms and *E. coli* (Fig. 3c, d). Considering that 28% and 12.9% of the tubewells were found contaminated before and after decontaminating the mouth of the tubewells, respectively (Fig. 3b–d), it can be inferred that greater numbers of the aquifers are safe. As shown in Fig. 3e, it appears that people do not adopt hygienic measures and contaminate the mouth of the tubewell in various ways, e.g., by inserting water pot used in the latrine, rubbing hands against the tubewell hose after coming from the latrine, etc. Figure 3f shows that the rough metal surface of tubewell mouth might be favorable for bacterial biofilm formation as reported by others [18]. The contamination that appeared after decontaminating the mouth of the tubewells might be the contamination of the aquifer or coming from other ways such as cracked pipe and/or loose-joints. It should also be considered that through burning alone, the mouth of the tubewells could be decontaminated, but the interior of the tubewell might remain contaminated. That is why all of the 12.9% tubewells that were found contaminated even after decontaminating the mouth of the tubewells might not entail contamination of aquifer(s).

We have tested *E. coli*, and faecal coliforms and in light of the observations could not rule out other sources of contamination such as by livestock, runoff, and compromise of well integrity, etc. To ensure contamination by only human activity, species analysis of indicator bacteria could help to rule this in or out. Our data suggest that 87% of the aquifers are not contaminated and we can assume that proper hygienic awareness, practices, and decontamination of the mouth of the tubewell at regular intervals might serve as possible interventions to provide safe drinking water.

### Aquifer contamination reduced with time

**Fig. 4:**
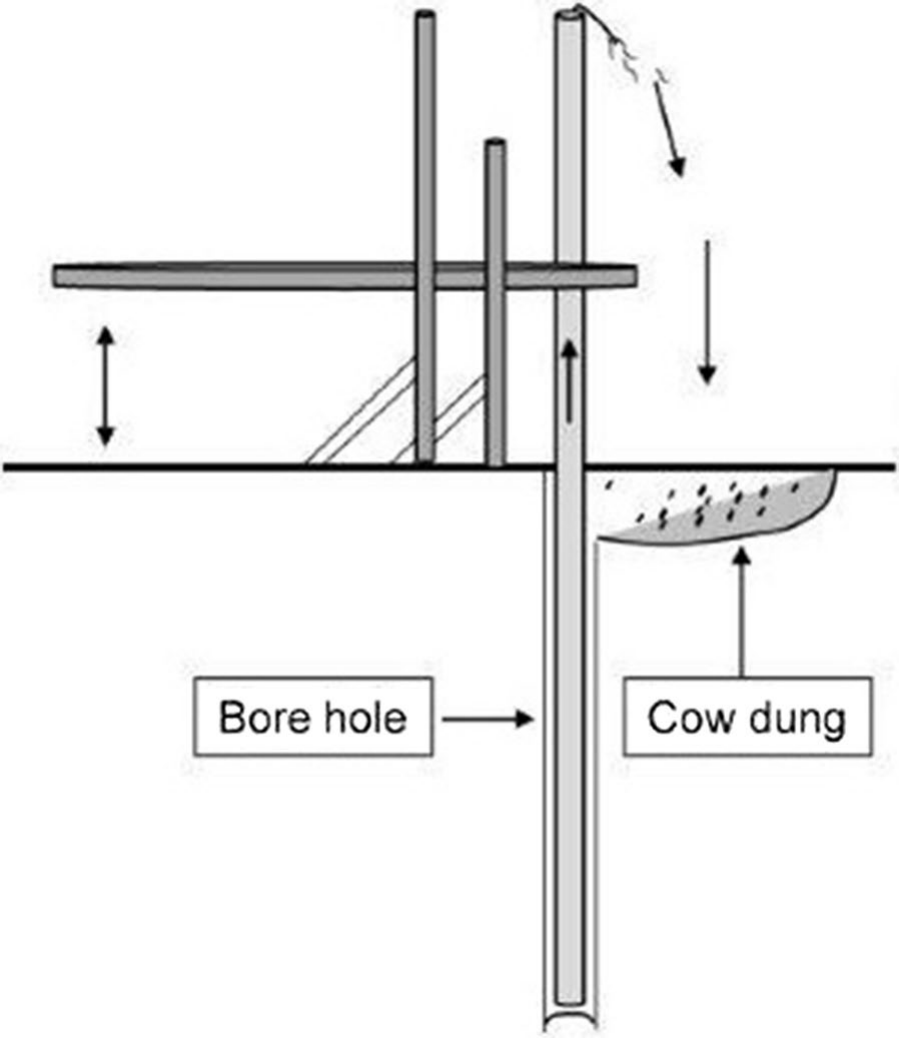
Schematic diagram showing the use of cow dung during the installation of tubewell

We have shown that most of the aquifers in the area of the camps are safe, but there were some possibilities of aquifer contaminations (n = 14, Table 3). The possible source of these contaminations might be due to the practice of the use of cow dung at the time of installation (Fig. 4) of the tubewells. The contamination might also happen as safe distances between shallow tubewells and latrines [19, 20] are not maintained in the Rohingya camps due to the heavy density of population. Water–cow dung mixture is usually employed to stabilize the walls of the borehole during drilling of the tubewell pipes (Fig. 4) possibly to reduce cost of tubewell installation and as an alternative of high cost materials such as bentonite clay [21]; also, there is a local concern that tubewell’s underground filter could be blocked by bentonite clay. We assumed that the contamination from cow dung might have been reducing over time, and as shown in Table 3; we observed that the contamination of water sources reduced after 1 month. This might be due to the survivability duration of faecal coliforms and *E. coli* in the deep aquifer and also reduction through washing out during purging of the tubewell water. The tubewells, of which the contamination was not found to be at baseline even after 1 month, were subsequently replaced by new tubewells. Water from these new tubewells were checked and found free of contamination.

**Table 3:** Arithmetic mean of contamination of same samples collected between 1 month intervals

	Types	First time sampling	Second time (after 1 month)
Faecal coliforms	Before (n = 14)	18,593	6382
	After (n = 14)	13,795	5428
	Household (n = 28)	15,948	7750
*E. coli*	Before (n = 14)	3592	2210
	After (n = 14)	2668	2233
	Household (n = 28)	715	528

### Risk categories of *E. coli* contaminations

**Fig. 5:**
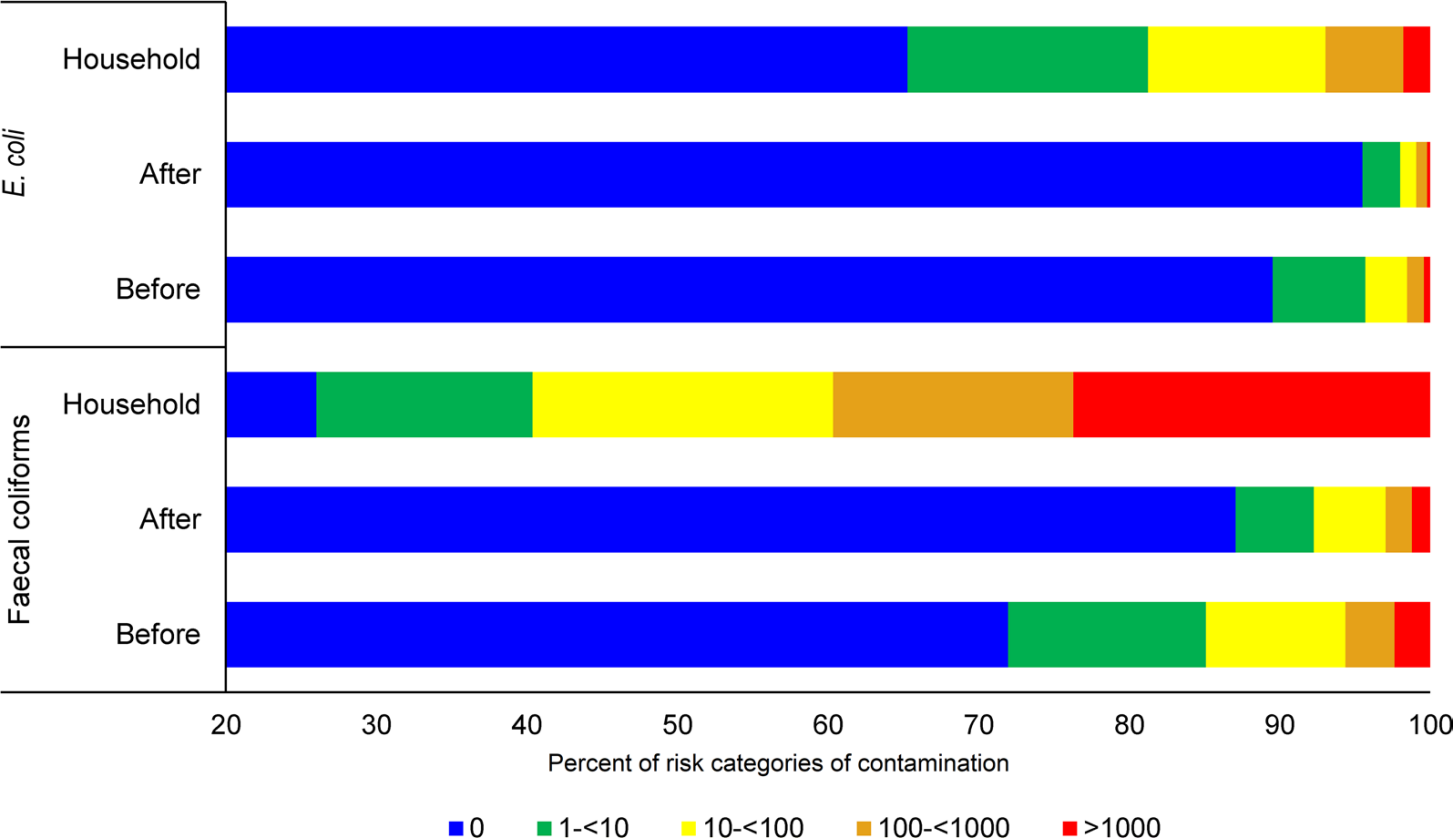
Risk categories of contamination. WHO (2011) has developed a classification and color-code scheme for *E. coli* colonies per 100 mL water sample which are (i) in conformity with WHO guideline—blue (0/100 mL); (ii) low risk—green (1–< 10/100 mL); (iii) intermediate risk—yellow (10–< 100/100 mL); (iv) high risk—orange (100–< 1000/100 mL); (v) very high risk—red (> 1000/100 mL)

Classification of risk categories of faecal coliforms and *E. coli* were also carried out to understand the water quality in Rohingya camps according to the WHO [7] classification and color-code scheme for *E. coli* colonies per 100 mL water sample. The distribution of *E. coli* and faecal coliforms according to some risk categories are presented in Fig. 5. As discussed earlier, the percentage of contaminated water in POU cases was higher than that of source water. In case of *E. coli* contamination, percentage of uncontaminated water in the source reduced from 89.5 to 65% in POU water (Fig. 5). In the case of faecal coliforms, that percentage of uncontaminated water decreased from 71.97 to 26.03% (Fig. 5). We observed that lower risk category of source water shifted to higher risk category in the POU water and this trend was observed for both faecal coliforms and *E. coli*. Of note, it was observed that a number of source water samples moved to the highest risk category in POU water for both faecal coliforms (75 to 1487) and *E. coli* (13 to 111). On the other hand, it was observed that after decontaminating the mouth of the tubewell, about 87% tubewells were found free of contamination and number of tubewells in all the risk categories decreased (Fig. 5). Our findings suggest that periodic decontamination preferably by burning of the mouth of the tubewells might be a possible way of intervention. Treatment and safe storage of household water from contaminated sources and safe storage of water from non-contaminated sources are important to reduce diarrhea outcomes [22, 23]. Nevertheless, treatment of household water and safe storage remains a major challenge for the concerned people or organizations to ensure safe drinking water supply [24, 25]. Our findings suggest that the POU water moved to the higher risk category as compared to source water and this might be due to lack of awareness of hygienic practices which resulted in secondary contamination.

Given the enormity and cross-sectional nature of our study, there might be quite a few limitations to be considered. First of all, we have collected water samples only once from tubewells and households. Although our sample size was big enough to statistically nullify outliers and we have maintained the cold chain strictly along with quick transportation of samples via air, the results of the test might have been slightly affected by the fact that the sampling site and the test laboratory were almost 400 km apart. There were some duplications during the collection of tubewell samples and we have excluded those from the analysis. There could be a little possibility that sample collectors may have inappropriately collected some of the water samples in the households, which might have affected our capacity to precisely determine drinking water quality parameters. To minimize the sampling errors, we have conducted extensive sessions of training and provided instruction sheets to the sample collectors on water sampling techniques before starting the project. Moreover, to ensure quality control, every sample collector collected a field blank and field duplicate sample and the lab microbiologists tested those samples along with lab duplicate and lab blank samples. The ANOVA test reveals that the count variation between original and duplicate samples (both field duplicate and lab) was not significant at a 95% confidence interval.

## Conclusion

Despite the limitations and challenges faced, this is the first study of water quality assessment in the Rohingya camps involving almost half of the total drinking water sources. Our findings demonstrate that almost all of the water samples collected after decontaminating the mouth of the tubewells were free from faecal contamination, and indicate that most of the underground acquifers are safe and we can assume that contaminations are mainly secondary. Secondary contamination might occur during collection and storage of water due to inadequate knowledge and lack of personal and domestic hygienic practices which needs to be studied. Therefore, necessary measures should be taken to build up awareness of proper hygienic practices and the contaminated household water should be treated by a suitable method to provide safe water.

## Methods and materials

### Study setting

**Fig. 6:**
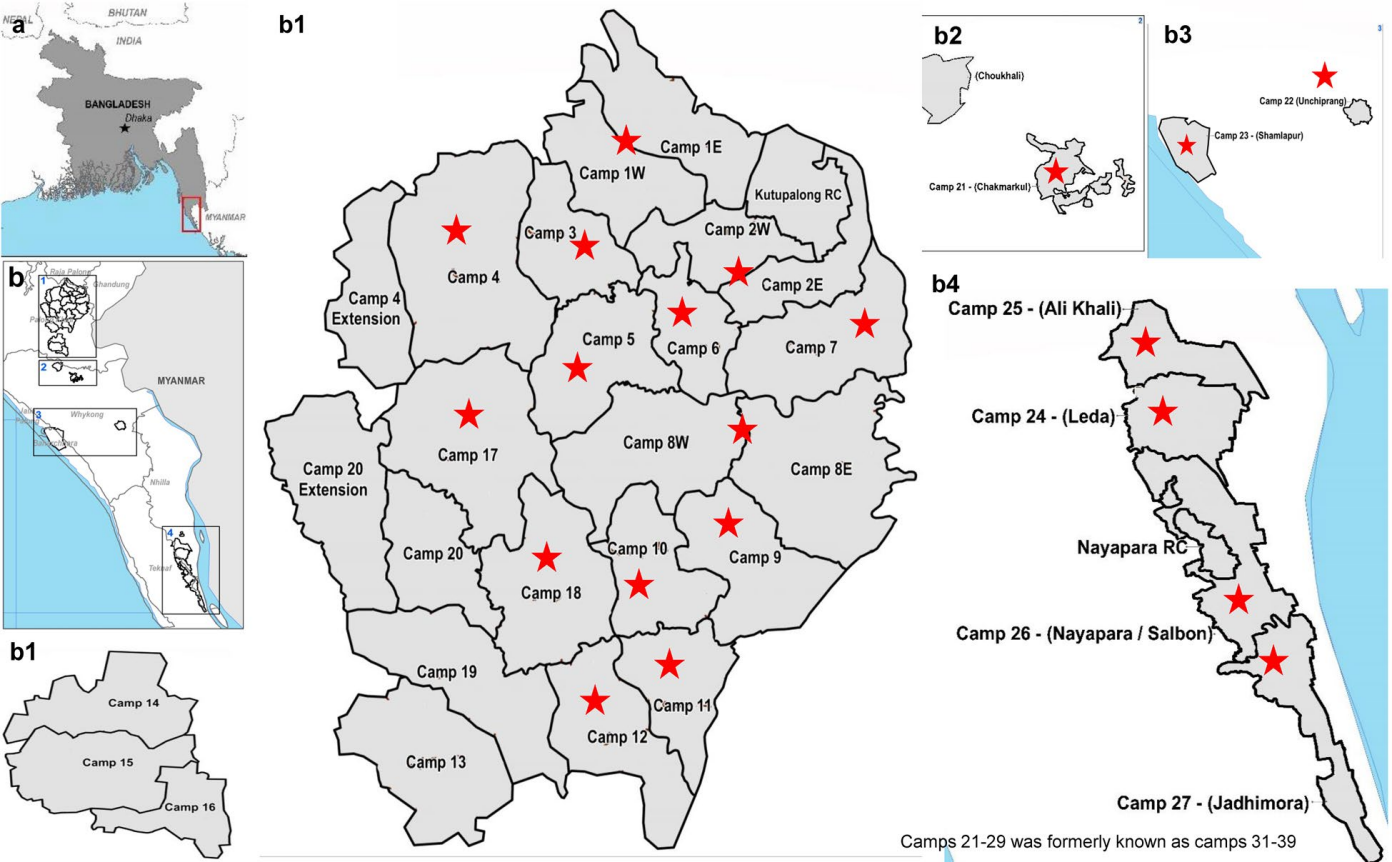
Map of Rohingya camps in Cox’s Bazar, Bangladesh. **a** Location of Rohingya camps in the map of Bangladesh (inset). **b** Location of Rohingya camps in Cox’s bazar. **b1**–**b4** correspond to detailed view of the location map(s) from **b** inset 1, 2, 3 and 4, respectively. Stars denote the camps from where the drinking water samples were collected (Source: OCHA/ReliefWeb https://reliefweb.int/sites/reliefweb.int/files/resources/71539.pdf) Accessed 19 October, 2019)

The study was conducted in 21 Rohingya camps out of the 32 camps present in the Cox’s Bazar district of Bangladesh, from February to September 2018. The camps were selected randomly. Locations of Rohingya camps in Cox’s Bazar, Bangladesh as well as the sampling areas are shown in Fig. 6.

### Mapping of tubewells

The locations of the tubewells in the Rohingya camps in Cox’s Bazar were identified with the assistance of volunteers of the WASH partners of unicef. Each tubewell was labeled with a unique ID (which was generated according to the national guidelines). A unique ID was written, in each case, at two places on the tubewells with water-resistant permanent markers of two different colors. At the same time, GPS locations of the tubewells, as well as photographs focusing the labels of the tubewells, were taken. This procedure was followed to avoid any duplicate sampling due to the high density of tubewells in the Rohingya camps.

### Collection of samples

From every selected camp, about 50% of total tubewells were sampled. Five hundred milliliter of water was collected from each point of source and POU for the purpose of analysis, using sterile wide mouth plastic bottles (Nalgene, USA) following standard procedures [26]. POU water was collected directly from the container (glass, mug, bottle, etc.) that was used for drinking. Upon collection, samples were placed in cool boxes with sufficient amount of ice packs and transported to the Laboratory of Environmental Health (LEH) of icddr,b, Dhaka by air, maintaining the temperature at 4 to 10 °C, and the samples were processed within 24 h of collection. Cold chain and processing within 24 h of collection were maintained to preserve the microbiological quality of the samples.

### Field blank/duplicate sample collection

Sample collectors carried a ‘field blank’ everyday as a negative control to observe field sampling conditions. The field blank was autoclaved water in a sterile container provided from the lab. The sample collectors just carried the container with sterile water in their cool box and opened the cap of the container once in the field and then closed the cap of the container tightly afterwards and sent back the field blank sample together with other samples to the laboratory for processing.

Randomly, a duplicate sample for one of every 20 samples was also collected. These duplicate samples were collected for both the source and the POU water samples. The same sampling procedure was followed for duplicate samples except that they were marked ‘DUP’ on the label in addition to the normal labeling.

### Lab blank/lab duplicate sample processing

Each day the lab expert(s) had to test a ‘lab blank’ as a negative control to assess lab testing conditions. The lab blank was autoclaved water kept in a sterile container of the same type that was provided for the field and was processed with other samples using the same procedure.

The lab expert(s) also had to duplicate the test from one of every twenty samples, randomly. These lab duplicate tests were done for all types of water samples including sources and POU water. These results served as the indicator of the accuracy of lab testing procedures.

### Sample size

We have collected four types of samples from field, namely, (i) samples before decontamination of the mouth of the tubewell (B), (ii) samples after decontamination of the mouth of the tubewell (A), (iii) two household-POU samples (H) from each corresponding tubewell and (iv) a field blank sample with each day sampling (FB). We have also collected around 5% duplicates of all the samples except field blank samples. Field blank samples were collected by each collector once in a collection day. A total of 14,522 different types of samples were collected and tested.

### Quality control

Due to being a cross-sectional study, we have collected samples only once from every source by following robust quality control measures such as the collection of 5% field duplicates from all types of water sources and collection of a field blank sample for every batch of the sample. Furthermore, we have tested around 5% of the samples as lab duplicates and used lab blanks with every batch of samples. Sample selection and rejection criteria were also determined and followed strictly. The cold chain was maintained strictly by using cock-sheet packaging system filled with enough ice packs and samples were only accepted if the bottles were sealed and the pack temperature was between 4 and 10 °C.

### Laboratory analysis

*Escherichia coli* and faecal coliforms were enumerated as described elsewhere [27, 28]. In brief, 100 mL water sample was filtered through a 0.22 μm cellulose-nitrate membrane (Sartorius Stedim Biotech GmbH, Goettingen, Germany) through a Millipore manifold filtration system (EZ-Fit™ Manifold, Merck KGaA, Darmstadt, Germany) in which microorganisms are retained on the membrane surface. Membrane filter was transferred on a selective culture medium (mTEC for *E. coli* and MFC for faecal coliforms) in a petri plate and incubated at 35 ± 0.5 °C for 2 h followed by further incubation at 44.5 ± 0.2 °C for approximately 22–24 h for *E. coli* and 44.5 ± 0.2 °C for 20 ± 2 h for faecal coliforms. The colonies developing magenta color on mTEC and blue color on MFC media were counted as *E. coli* and faecal coliforms with unaided eye, respectively. Dilutions were made where the colonies were too numerous to count. Plates were counted as soon as they were removed from the incubator.

### Statistical analysis

The Statistical Package for the Social Sciences (SPSS) was used for the analysis of experimental data (SPSS version 20.0, IBM). Descriptive statistics, ANOVA and odds ratio calculations were main tools of statistical analysis used in the study and are represented in either graphical or tabular form. Regression analysis was performed to identify linear relationship and also a comparison of the calculated r-values was done in different confidence levels using the standard statistical table. In hypothesis testing, the P-value is compared in 5% and 1% level of significance. As this was a cross-sectional study and that all the samples were collected and analyzed once, the triplicate data based statistical analysis could not be performed.

## Supplementary information

**Additional file 1: Table S1.** Number of three different types of water samples collected from 15 Rohingya camps (50% of all functional tubewells of respective camps).

## Data Availability

The datasets used and/or analyzed during the current study are available from the corresponding author on reasonable request.
