# Supplementary material for: Occurrence of Escherichia coli and faecal coliforms in drinking water at source and household point-of-use in Rohingya camps, Bangladesh
Source: Gut Pathog. 2019 Nov 1;11:52. doi: 10.1186/s13099-019-0333-6 (PMC6824040; doi:10.1186/s13099-019-0333-6)
Supplement: Supplementary file 1 — Additional file 1: Table S1. Number of three different types of water samples collected from 15 Rohingya camps (50% of all functional tubewells of respective camps). [file 13099_2019_333_MOESM1_ESM.docx]

**Table S1 Number of three different types of water samples collected from 15 Rohingya camps (50% of all functional tubewells of respective camps)**

| **Camp No.** | **Before** | **After** | **Household** | **Total** |
| --- | --- | --- | --- | --- |
| 01 | 668 | 668 | 1326 | 2662 |
| 02 | 258 | 258 | 510 | 1026 |
| 03 | 295 | 295 | 505 | 1095 |
| 04 | 148 | 148 | 296 | 592 |
| 05 | 97 | 97 | 194 | 388 |
| 06 | 94 | 94 | 188 | 376 |
| 07 | 179 | 179 | 355 | 713 |
| 08 | 326 | 326 | 652 | 1304 |
| 09 | 274 | 274 | 548 | 1096 |
| 10 | 183 | 183 | 368 | 734 |
| 11 | 249 | 249 | 498 | 996 |
| 12 | 136 | 136 | 272 | 544 |
| 18 | 118 | 118 | 238 | 474 |
| 31 | 58 | 58 | 116 | 232 |
| 34 | 32 | 32 | 62 | 126 |
| **Total 15 camps** | **3115** | **3115** | **6128** | **12358** |
